# Work-From-Home During COVID-19 Lockdown: When Employees’ Well-Being and Creativity Depend on Their Psychological Profiles

**DOI:** 10.3389/fpsyg.2022.862987

**Published:** 2022-05-09

**Authors:** Estelle Michinov, Caroline Ruiller, Frédérique Chedotel, Virginie Dodeler, Nicolas Michinov

**Affiliations:** ^1^Laboratory of Psychology: Cognition, Behavior and Communication (LP3C, UR 1285), Department of Psychology, University of Rennes, Rennes, France; ^2^Laboratory CREM (UMR CNRS 6211), Graduate School of Management, University of Rennes, Rennes, France; ^3^Laboratory GRANEM (UR 7456), Graduate School of Management, University of Angers, Angers, France

**Keywords:** COVID-19, work-from-home, well-being, creativity, preference for solitude, big-five dimensions

## Abstract

With the COVID-19 pandemic, governments implemented successive lockdowns that forced employees to work from home (WFH) to contain the spread of the coronavirus. This crisis raises the question of the effects of mandatory work from home on employees’ well-being and performance, and whether these effects are the same for all employees. In the present study, we examined whether working at home may be related to intensity, familiarity with WFH, employees’ well-being (loneliness at work, stress, job satisfaction, and work engagement) and creativity (‘subjective’ and ‘objective’). We also examined whether the psychological profile of employees, combining preference for solitude and associated personality variables from the Big Five, may influence the effects of WFH. The data were collected *via* an online survey from November 13th to December 15th 2020 among 946 employees from various organizations during the second lockdown in France. In addition to identifying two distinctive psychological profiles for employees having to WFH, results revealed that those with a “Solitary” profile reported higher loneliness at work, higher levels of stress, and lower levels of job satisfaction and work engagement than those with an “Affiliative” profile. It was also found that employees with a “Solitary” profile perceived themselves as less creative and produced objectively fewer ideas than individuals with an “Affiliative” profile. The present study suggests the necessity to distinguish the profiles of teleworkers and to offer a stronger support for the less affiliative employees when working from home.

## Introduction

The COVID-19 pandemic has caused unprecedented challenges to health systems and the global economy. Companies and institutions have had to change their work organization requiring employees to adapt quickly to new constraints. The rules of social distancing and the successive lockdowns put in place by governments in different countries have obliged employees to work at home. For the first time in modern history, employees had to telework every day from home, without being prepared for it, without specific arrangements or formal contracts from their companies. During the COVID-19 pandemic, “remote working has become the “new normal” almost “overnight” (Wang et al., 2021, p. 17), forcing people to work from home without taking into consideration their preferences, abilities, personality, and the nature of their jobs (Wang et al., 2021). The COVID-19 pandemic, and the related lockdowns, are thus a novel home-based work context that differs from literature studies on the effects of telework before the pandemic.

As previously defined by Taskin and Devos (2005), teleworking normally refers to carrying out a professional activity, partly and sometimes fully at a distance and requiring the use of information and communication technologies (Allen et al., 2015; Vayre, 2019). To the extent that it breaks with a certain unity of time, place and action, telework involves a qualitative shift away from traditional forms of centralized social organization and toward a more diffuse, fragmented and emergent set of social relations (Ajzen and Taskin, 2021). The nature of teleworking during the pandemic and containment periods was different from this previous definition of telework before COVID-19. Working from home (WFH) during the COVID-19 pandemic was an enforced decision obliging a significant proportion of the working population to work at home extensively (e.g., full-time during the first lockdown). Employees had to comply with governmental restrictions to limit the risk of infection or spread of coronavirus. They had little time to plan and prepare the telework, they may have had a restricted physical workspace at home and taken on multiple roles, and this was combined with restricted freedom to travel and limitations on social contacts (de Macêdo et al., 2020; Waizenegger et al., 2020). Some authors refer to this new form of teleworking as “Mandatory Work From Home” (Kniffin et al., 2021, p. 65).

As the COVID-19 pandemic has required mandatory WFH solutions to be adopted massively, some scholars recently began to conduct empirical studies to understand the effects of WFH on employees’ job satisfaction, performance, and well-being (Galanti et al., 2021; Graham et al., 2021; Guler et al., 2021; Stempel and Siestrup, 2022). The first findings show that not all remote workers are the same, pointing out differences such as demographic variables (Pathak et al., 2021) or remote work acceptance and beliefs (Donati et al., 2021), which highlights the need to improve understanding of the different WFH experiences or profiles.

While remote work can benefit employees regarding a number of behaviors such as work flexibility, time saving and reduction in transportation time, its impact on creativity has been up for debate because WFH likely offers fewer opportunities to talk with colleagues and stimulate creative thinking, for example to find novel ideas to conduct an organizational project. The originality of this research is to focus not only on the employees’ job satisfaction and well-being during WFH, but also on ‘subjective’ and ‘objective’ creativity. Indeed, some recent research has highlighted that there is a positive relationship between creativity and individuals’ well-being, considering creativity as a useful strategy to cope with the COVID-19 pandemic (Tang et al., 2021). To our knowledge, no research has examined extensively teleworkers’ creativity during lockdown, except in a recent study measuring ‘subjective’ professional creativity through self-report measures by a questionnaire (Mercier et al., 2021). It revealed no differences on ‘subjective’ creativity among teleworkers during lockdown compared to employees who continued to work at their usual worksite. This led us to focus on the role of creativity, and to conduct a study to fill the aforementioned research gaps by administrating an online survey from November 13th to December 15th 2020 among 946 employees from various organizations working at home during the second lockdown in France. The present study aimed to examine whether employees’ well-being and creativity in a WFH context during the second lockdown would depend on their psychological profiles, and not only on demographics or the WFH experience. In such a context, some employees can be considered at risk depending on their preference for solitude and personality variables related to the Big Five dimensions.

### The Effects of Telework on Employees’ Well-Being and Performance

The literature on the effects of telework outside the COVID-19 pandemic has revealed contradictory findings on employees’ well-being and job performance, either positive or negative (Gajendran et al., 2015; Boell et al., 2016; Charalampous et al., 2019; Golden and Gajendran, 2019; Vayre, 2019; Wang et al., 2019, 2020). Some studies have examined the positive effects of telework and identified that teleworkers are less interrupted and have more concentration on the task (Vittersø et al., 2003; Windeler et al., 2017), and all these factors contribute to increased job satisfaction, employees’ well-being and job performance (Gajendran et al., 2015; Delanoeije and Verbruggen, 2020). It can be noted that studies on the effects of telework have focused mainly on well-being and very few on performance. In a field study (Vega et al., 2015), teleworking was also found to have positive implications on ‘objective’ creativity (e.g., finding an innovative solution in the context of urban development). Similarly, in an experiment aiming to examine differences of performance between repetitive and creative tasks performed remotely, Glenn Dutcher (2012) showed that teleworkers increased their performance in creativity tasks only (e.g., finding as many unusual uses for ordinary objects as possible). Some explanations have been provided as to why employees are creative in remote working, such as greater concentration on the tasks (Biron and van Veldhoven, 2016), and thinking alone and independently, two necessary conditions for producing novel ideas (Nouri et al., 2015). Teleworking also increases the employee’s sense of control over their working time, with greater flexibility in the way they organize their work (Kossek et al., 2006), less travel time and thus overall a better work-life balance (Golden et al., 2008; Anderson et al., 2015; Grant et al., 2019). The sense of autonomy is higher and increases motivation, organizational commitment, and job satisfaction (Charalampous et al., 2019; Grant et al., 2019). Overall, telework can be seen more as a resource, both for the company to support job performance (Gajendran and Harrison, 2007), and for the workers to enhance their work engagement (Masuda et al., 2017) and well-being (Anderson et al., 2015).

Other studies have examined the negative effects of telework, and at least three risk factors for employees have been identified. Firstly, it increases pressure on teleworkers regarding constant connectivity and responsiveness. It results in intensifying work, distracting from the family members and creating work-family conflict (Hammer et al., 2005; Allen et al., 2015; Derks et al., 2016; Grant et al., 2019). Telework can also lead to higher stress and depression (Song and Gao, 2020; Afonso et al., 2021), technostress (Suh and Lee, 2017; Vayre and Vonthron, 2019; Ghislieri et al., 2022), or workaholism (Vayre, 2019; Song and Gao, 2020). Secondly, due to a greater physical distance from teamwork, a feeling of social isolation, alienation and loneliness can emerge (Zhou, 2018; Charalampous et al., 2019). The need-to-belong theory (Baumeister and Leary, 1995) can explain how teleworkers’ physical and psychological isolation may reduce their affective connections to their coworkers (Wang et al., 2020). There can also be a lack of support and visible leadership, and there may be less social interaction when isolated and detached from the workplace. The perceived distance of teleworkers from their team can have a negative impact on performance and can increase job dissatisfaction (Golden et al., 2008; Ozcelik and Barsade, 2018). Remote employees should initiate frequent communications with their coworkers, and organizations have to provide adequate social support by providing an enabling organizational context (Ruiller et al., 2019). However, although remote workers perceive themselves as having more autonomy and more flexibility thus reducing their stress, at the same time, they can feel socially isolated and disconnected from their peers and supervisors, leading to an ambivalent impact on well-being (Ruiller et al., 2019; Pulido-Martos et al., 2021). Finally, another risk of telework concerns the difficulty for the teleworker to find his or her own work organization and to motivate himself or herself in the absence of formal teamwork. The use of information and communication technologies also leads to counterproductive behavior, such as cyberslacking, i.e., the voluntary use of the Internet for non-work-related purposes during working hours (O’Neill et al., 2014b).

To summarize, the effects of telework on employees seem to be inconsistent or even divergent. Several factors can explain the inconsistent effects of telework (Oakman et al., 2020): the intensity of telework, characteristics of the telework environment, the nature of work (e.g., task interdependence, autonomy), the organizational culture and leadership management, the technical and social supports (e.g., supervisor support, colleagues support), social relationships external to work (e.g., family support, friends support) and the nature of outcomes studied (e.g., job satisfaction, mental health, self-rated or objective performance, productivity or creative performance). As the COVID-19 pandemic imposed telework on all, it also seems important to know the profiles of teleworkers, notably to identify those at risk, in order to manage better the implementation of WFH. To date, we ignore which psychological profiles of employees benefit or suffer from teleworking. Individual differences or personality characteristics of teleworkers could contribute to explaining the inconsistent effects observed, as “one size does not fit all” (Charalampous et al., 2019, p. 69).

### The Moderating Role of Individual Differences

Although most studies have focused on situational or organizational variables that influence the effects of telework, some research has tried to identify individual differences that might influence the acceptability and the effects of telework on employees’ well-being and performance (Allen et al., 2015; Anderson et al., 2015; Donati et al., 2021; Wang et al., 2021).

Concerning the effects of personality traits on employees’ well-being during telework, some personality variables related to the Big Five, such as agreeableness, openness to experience, or consciousness appear to be positively related to telework acceptability and employees’ well-being, while mixed-results were found for extraversion and neuroticism. A high level of openness to experience would thus moderate the positive correlation between telework and positive emotions, making it more pronounced (Anderson et al., 2015). The authors explain this by the creative, curious, eager for novelty and change aspects of this personality type, which would allow employees to cope better with the many adaptations and flexibility that telework requires (Anderson et al., 2015). During WFH conscientious people are able to set non-immediate goals, and are well organized, self-disciplined and autonomous at work (Wilmot et al., 2019; Kniffin et al., 2021). Concerning the effects of extraversion and neuroticism traits on employees’ well-being during WFH, the findings are not consistent. In general, extraversion is associated with lower work-stress and higher job satisfaction (Udayar et al., 2020). Thus, individuals who are more sociable and attracted to developing relationships with others tend to report more adaptability to telework and less deleterious effects of social isolation (O’Neill et al., 2009; Anderson et al., 2015; Wilmot et al., 2019). Recent studies during the COVID-19 pandemic revealed that extraversion is associated with more active seeking of socioemotional support and adaptative strategies of coping (Volk et al., 2021). On the contrary, a study conducted in Norway with police officers did not find a significant association between extraversion and general satisfaction with the home-office arrangement (Langvik et al., 2021). Another study with Canadian adults recently showed that both higher neuroticism and extraversion scores were associated with high levels of stress during the pandemic (Liu et al., 2021). In a recent longitudinal study, Entringer and Gosling (2021) showed that loneliness increased significantly compared to pre-pandemic levels, and to a greater extend for women and younger people, but also for extraverted, neurotic and conscientious individuals. Thus, the effects of extraversion on the acceptability of WFH and its effects on employees’ mental health need further investigation. Concerning the effects of neuroticism on teleworkers’ well-being, the results are also inconsistent. Some studies suggest that the deleterious effects of telework on mental health are greater with higher levels of neuroticism (Wilmot et al., 2019). Employees who are more emotionally stable are more able to establish good interpersonal relationships with others and to capitalize on positive emotions. By contrast, other studies suggest that individuals high on neuroticism reported positive attitudes toward remote work (Clark et al., 2012), possibly because they saw this as an opportunity to exert less effort and as a way to avoid difficult relationships with others in the workplace. Thus, studies on the effects of personality traits on attitudes to telework and on employees’ well-being do not always give consistent results.

Concerning the effects of personality traits on employees’ performance during telework, very few studies have investigated the influence of certain personality traits or individual differences (O’Neill et al., 2009; Allen et al., 2015; Wang et al., 2021). However, these studies have generally not focused on ‘objective’ performance of employees, or notably on creativity, except in recent studies conducted during the COVID-19 pandemic which reveal that personality and individual difference related to the preference for solitude (Burger, 1995) may moderate the effects of social isolation on performance on creative tasks during lockdown (Michinov and Michinov, 2021). In a study carried out in France during the first lockdown, Michinov and Michinov (2021) showed that the level of loneliness, stress and anxiety varied according to the level of preference for solitude and certain traits related to the Big Five (extraversion, neuroticism, openness). Individuals with an “Affiliative” profile (i.e., individuals with low preference for solitude, extraverted and emotionally stable) as well as an “Emotionally Unstable Lonely” profile (i.e., individuals with high levels of neuroticism and openness, and moderate level of extraversion) expressed high stress and anxiety during lockdown, and the latter generated a greater number of creative ideas than the former. By contrast, those with an “Emotionally Stable Lonely” Profile (i.e., individuals with a high level of preference for solitude, low levels of extraversion and neuroticism) expressed a lower level of loneliness during lockdown and performed better on a creativity task requiring a correct solution to be found through insight. However, these data have been observed on the general population, and deserve to be studied on a specific population of teleworkers.

### Objective of the Study and Hypotheses

Thus, it appears that not all people are equal when faced with social isolation and distance from peer groups, whether friendly or professional groups. Moreover, prior studies were conducted with individuals from the general population and did not specifically examine a sample of employees working from home during the COVID-19 pandemic. In this perspective, it seems necessary to extend research to: (1) examine the relationships between the intensity and familiarity with telework, employees’ well-being, employees’ creativity and personality variables; (2) identify psychological profile of employees at risk from social isolation during mandatory WFH, and (3) examine if distinct personality profiles affect employees’ well-being and creativity during mandatory WFH.

Based on research demonstrating the moderating role of personality variables on acceptability of telework and performance (O’Neill et al., 2009; Allen et al., 2015; Anderson et al., 2015; Donati et al., 2021; Wang et al., 2021), and the role of personality variables and individual differences on individuals’ mental health and creativity during lockdown (Entringer and Gosling, 2021; Götz et al., 2021; Liu et al., 2021; Michinov and Michinov, 2021; Volk et al., 2021), we expected that personal characteristics of teleworkers would affect well-being and creativity during mandatory WFH. More specifically, we could expect the effects of WFH to be related not only to the characteristics of WFH (experience or intensity) but also to teleworkers’ psychological profiles based on their preference for solitude and personality variables related to the Big Five dimensions (**Hypothesis 1**). Although it is difficult to determine *a priori* the psychological profiles that can emerge from a sample of teleworkers, based on a recent study by Michinov and Michinov (2021) on a wider population than those of employees, we could expect that “Affiliative” and “Emotionally Unstable Lonely” individuals should express higher loneliness at work and stress, but less job satisfaction and work engagement, and the latter should produce a greater number of creative ideas than the former those with an “Emotionally Stable Lonely” profile (**Hypothesis 2**).

## Materials and Methods

### Participants and Procedure

Participants completed an anonymous online survey using the LimeSurvey software (Limesurvey GmbH, Limesurvey GmbH), after having read the written consent form and explicitly agreeing to participate. The study was conducted in accordance with the Declaration of Helsinki. Ethical approval was granted by the Ethics Committee of the University of Rennes 2 (No 2021-030). There was no monetary or credit compensation. The survey was shared *via* social media from November 13th to December 15th 2020 during the second lockdown in France.

Participants were recruited *via* snowball sampling. The authors of the article and students from Masters in work and organizational psychology, and human resources management posted an advertisement to recruit participants on their social media profile and disseminated the advertisement directly to personal and professional contacts working in diverse occupations. This survey was entitled “My experience of teleworking,” and was addressed to teleworkers over 18 years old living in France. A total of 1,155 responses were collected, and 209 responses were deleted because of incomplete responses to the scales (108 returns with no response for the stress, well-being and creativity scales, 101 with no response for the creativity scales or socio-demographic data). The final sample therefore included the responses of 946 participants. The valid response rate (complete data) was thus 81.90%.

Participants were 73.7% females, and they had a mean age of 35.4 (SD = 11.5). The majority were married or with a partner (69.9%), 55.71% had no children, 13% one children, 24% two children, 6.6% three children, and 0.7% more than four children. Most of them had a bachelor’s or master’s degree (90.4%). Most of them had a full-time job (87.6%) and a permanent contract (74.9%). On average, their organizational tenure was 4.83 years (SD = 6.04). They came from diverse occupations, for example, banking, insurance, finance (13.3%), health and social work (11.6%), education (9.7%), the industrial sector (9.5%) and business services (9.5%). Among the participants, 48.2% worked in large organizations (300 or more employees), 18% in medium organizations (101–300 employees), 18% in medium-small organizations (21–100 employees) and 14.8% in small organizations (1–20 employees).

A majority of respondents were teleworking 5 days per week (49.2%), with the rest working 4 days (15.8%), 3 days (12.9%), 2 days (12.4%) or 1 day (6%) per week. Most of them reported that telework was not a chosen situation (76.8%) and 51% declared that telework had been formalized by a contractual agreement during the COVID-19 lockdown. Outside this period of lockdown, the experience of telework was varied among respondents (38.5% had never teleworked, 32.8% rarely, 21.4% often, 7.4% very often), and we distinguished between high and low experience teleworkers. The most common telework space was at home with a dedicated room or office (52.2%) and for 47.6% at home without a dedicated room. The equipment was provided entirely by the employer (47%) or only in part (37%). The vast majority of participants said they had help from colleagues or a helpdesk in case of technical problems (91.9%).

### Measures

The questionnaire consisted of brief measures to be administered in large online surveys which are known to have good validity and reliability across different samples. All the scales had already been largely used and validated in French, except for the loneliness at work scale. For this scale, the items were adapted using a back-translation procedure. The first author translated the scale. Masters’ students in Psychology and Organizational Management also reviewed each item of the questionnaire to ensure the face validity. A bilingual professional translator then checked the scale and ambiguous or complex terms were either removed or rephrased. The details of items used are presented in Supplementary Material.

#### Preference for Solitude

We used the French version of the Preference for Solitude Scale (PSS, Burger, 1995; Michinov and Michinov, 2021). This scale comprises 12 forced-choice statements. One option reflects a preference for solitude (coded 1) and the other a preference for being with other people (coded 0). Sample items include “I enjoy being around people vs. I enjoy being by myself” and “Time spend alone is often productive for me vs. Time spend alone is often time wasted for me.” The global score of preference for solitude was calculated from the sum of the 12 items. A confirmatory factor analysis indicated overall satisfactory fit indices for a one-factor structure, χ^2^(54) = 255, *p* < 0.001, TLI = 0.85, CFI = 0.88, SRMR = 0.04, RMSEA = 0.06. The internal consistency of this scale was satisfactory, α = 0.75.

#### Personality Variables

We used a 10-item short version of the Big-Five Inventory in French (Rammstedt and John, 2007; Courtois et al., 2020). The BFI-10 is designed for large-scale assessments with limited time resources. This scale was composed of 10 items that were selected as being the most representative of the five dimensions (Extraversion, Agreeableness, Conscientiousness, Neuroticism, Openness to experience), i.e., among the most saturated by the factor, with the constraint of having for each dimension one item formulated positively and another negatively. For example, for Extraversion, the item “Is sociable, extraverted” and the reverse item “Is reserved.” The response was reported on a five-point Likert scale, ranging from 1 (*disagree strongly*) to 5 (*agree strongly*). A confirmatory factor analysis indicated satisfactory fit indices for a five-factor structure, χ^2^(25) = 95.2, *p* < 0.001, TLI = 0.91, CFI = 0.95, SRMR = 0.03, RMSEA = 0.05.

#### Stress

Occupational stress was operationalized using a single-item of stress symptoms (Elo et al., 1992, 2003): “Stress means a situation in which a person feels tense, restless, nervous or anxious or is unable to sleep at night because his/her mind is troubled all the time. Do you feel this kind of stress these days?” The response was recorded on a five-point Likert scale ranging from 1 (*not at all*) to 5 (*very much*). This measure has been used in online surveys for a variety of occupations, corresponding to other measures of mental exhaustion (Elo et al., 2003) and has proven to be a valid measure of occupational stress by the National Institute for Health Research in France (Langevin et al., 2012).

#### Loneliness at Work

Loneliness was measured using a French adaptation of the loneliness at work scale (LAWS, Wright et al., 2006). This scale consisted of 16 items measured on a seven-point Likert scale, ranging from 1 (*strongly disagree*) to 7 (*strongly agree*). Items were for example: “I often feel isolated when I am with my colleagues,” “I often feel abandoned by my colleagues when I am under pressure at work.” Among the 16 items, seven were reversed and therefore were recoded (e.g., “There is someone at work with whom I can take my break if I want to”). Confirmatory factor analyses showed that a two-factor solution fitted better [χ^2^(103) = 812, *p* < 0.001, TLI = 0.90, CFI = 0.92, SRMR = 0.04, RMSEA = 0.08] than a one-factor solution [χ^2^(104) = 1025, *p* < 0.001, TLI = 0.88, CFI = 0.89, SRMR = 0.05, RMSEA = 0.10]. As in the study by Wright et al. (2006), there were two dimensions of loneliness at work: emotional deprivation with nine items (α = 0.92) and social companionship with seven items (α = 0.84).

#### Job Satisfaction

Single measures of affective well-being at work have often been used in online surveys (Gillet et al., 2013; Russell and Daniels, 2018). A single-item of job satisfaction was used: “Overall, I am satisfied with my work.” The response was reported on a 10-point Likert scale ranging from 1 (*not at all*) to 10 (*very much*).

#### Work Engagement

We used the French version of the nine-item work engagement scale (Schaufeli et al., 2006; Zecca et al., 2015). For example, the absorption item was: “I am completely absorbed by my work,” the dedication item was “I am passionate about my job” and the vigor item was “When I get up in the morning, I want to go to work.” The responses were reported on a seven-point Likert scale ranging from 1 (*never*) to 7 (*always/every day*). Confirmatory factor analyses showed that a three-factor solution fitted better, χ^2^(24) = 367, *p* < 0.001, TLI = 0.91, CFI = 0.94, SRMR = 0.08, RMSEA = 0.11, than a one-factor solution χ^2^(27) = 1276, *p* < 0.001, TLI = 0.71, CFI = 0.78, SRMR = 0.08, RMSEA = 0.22. Reliabilities were satisfactory (α = 0.84 for vigor, α = 0.79 for absorption, and α = 0.90 for dedication). For each dimension, an average score was calculated from three items.

#### Subjective Creativity

Self-rated creative performance was measured with the three-item creative self-efficacy scale (Tierney and Farmer, 2002). A sample item is: “I have confidence in my ability to solve problems creatively.” The responses were reported on a seven-point Likert scale ranging from 1 (*strongly disagree*) to 7 (*strongly agree*). The overall perceived creativity score was obtained by averaging the scores for each item. The internal consistency of this scale was satisfactory, α = 0.87.

#### Objective Creativity

An open-ended question at the end of the questionnaire was used to measure ‘objective’ creativity from two indicators of divergent thinking: fluency and originality. Participants were given this instruction adapted from the study of Vega et al. (2015): “Imagine you are a city planner for a new major city that is being built. One of the major concerns of the new residents is the traffic that is expected with the new businesses and population moving into the area. Describe a solution that will minimize the amount of traffic that people will experience. You have 5 min to generate as many ideas as possible.” The number of ideas given by each participant (fluency) was counted independently by three coders. Cohen’s Kappa was 0.92, which corresponds to a strong inter-coder agreement. The *fluency score* was based on the average of the three coders, and varied between 0 and 14 ideas. The originality of ideas was evaluated by three coders on a five-point Likert scale ranging from 1 (*low originality*) to 5 (*high originality*). Intra-Class Correlation (ICC) coefficient shows a satisfactory agreement between the three coders (ICC = 0.77, *t* = 4.32, *p* < 0.0001). The *originality score* was calculated from the mean of idea originality evaluated by the three coders, and ranged from 0 to 5. The fluency and originality scores were positively correlated (*r* = 0.51, *p* < 0.001), as is commonly observed on divergent thinking.

#### Control Variables

Previous studies have shown that age, gender, number of children, WFH experience and WFH intensity can influence remote workers’ performance and well-being (e.g., Kossek et al., 2006; Wang et al., 2021). Thus, we controlled for these variables when testing our model.

### Analytic Approach

The data analyses were performed using Jamovi (The Jamovi Project, 2021) and the ‘Machine Learning’ module (Clustering) of the JASP software (JASP Team, 2022) from the datasets available in our OSF project (Michinov et al., 2021). The descriptive statistics of the quantitative variables were reported as frequencies and percentages. Relationships between study variables were examined with Pearson’s correlations. Multiple regression analyses were conducted to examine the relative contributions of control variables, preference for solitude and personality variables on indicators of well-being (i.e., loneliness at work, stress, job satisfaction and work engagement) and creative performance (i.e., ‘subjective’ and ‘objective’ creativity).

To explore the data thoroughly, we used a person-centered approach to identify the psychological profiles of employees which fit WFH. Since we could not specifically predict the type of teleworker profiles during this period of lockdown, we chose to use exploratory data clustering techniques with a combination of hierarchical (Ward’s method) and non-hierarchical (K-means analysis) methods on a standardized dataset without outliers (Hair et al., 2010). These common methods have been used in several studies with a large number of data sets. It is also important to assess interpretability and theoretical meaningfulness of the latent profiles when determining the optimal solution. For this, we used common graphical representations (i.e., the dendrogram of cluster analysis, elbow method, and t-SNE cluster plots) when determining the optimal number of clusters.

To determine whether identified clusters significantly differed on the preference for solitude and personality variables (extraversion, openness, conscientiousness, agreeableness and neuroticism), multivariate analyses of variance (MANOVA) were conducted. Finally, we examined the effects of the profiles on well-being and creative performance measures with analysis of variance (ANOVAs). *Post-hoc* tests were corrected for multiple comparisons with the Bonferroni procedure when appropriated. The final sample for analyses consisted of 946 participants. A sensitivity power analysis was conducted using G*Power (Faul et al., 2007). The sample size gave us a power of 95% to detect effects of at least Cohen’s *f*^2^= 0.02 for the linear multiple regression, 0.07 for the MANOVA, and 0.12 for the ANOVA.

To check the potential problem of common method bias when self-reported data is used, we performed some of the statistical remedies suggested by Podsakoff et al. (2003). First, we used Harman’s one-factor test (Fuller et al., 2016). We loaded all items of all measures into an exploratory factor analysis (EFA), using maximum likelihood extraction, and examined the solution. No single factor accounting for the majority of the covariance among measures emerged. We obtained a factor solution with the first factor explaining 20.4% of variance. As the first factor accounted for less than 50–60% of the variance among variables (Saris and Gallhofer, 2014), we can assume that there was no common method variance problem. Then, we conducted a CFA on the same dataset, in which all items loaded on a single factor. The solution found had mediocre fit indices: χ^2^(1080) = 10770, *p* < 0.001, CFI = 0.493, TLI = 0.470, RMSEA = 0.097, SRMR = 0.103. These preliminary statistical analyses suggest that common method bias was not a serious problem in this study. We also followed the methodological recommendations (Podsakoff et al., 2003) in building the questionnaire by assuring participants that their responses were anonymous and treated confidentially, by separating the blocks of measures from each other and by varying the response scales.

## Results

### Descriptive Statistics and Intercorrelations

Table 1 reports the values for means and standard deviations, skewness and kurtosis, and intercorrelations between study variables. All significant correlations are small (0.20), or moderate (0.50) in size according to Cohen’s criteria (Cohen and Cohen, 2003). The strongest correlations (>0.65) concern the relationships between the sub-dimensions of some scales (i.e., emotional deprivation and companionship, vigor, absorption and dedication). Moreover, some correlations indicate that the control variables (i.e., gender, age, number of children, WFH experience and WFH intensity) were related to the measured variables, and thus needed to be controlled for in the analyses. On the other hand, as expected, significant relationships were found between the variables related to preference for solitude and associated personality variables and measures of well-being and creative performance.

**TABLE 1:** Descriptive statistics and correlations of studied variables.

	*1*	*2*	*3*	*4*	*5*	*6*	*7*	*8*	*9*	*10*	*11*	*12*	*13*	*14*	*15*	*16*	*17*	*18*	*19*	*20*	*21*
1. Age	–																				
2. Gender	−0.129***	–																			
3. With children	0.708***	−0.084*	–																		
4. WFH experience	0.142***	–0.061	0.098**	–																	
5. WFH intensity	−0.086**	–0.058	−0.097**	0.232***	–																
6. Pref. solitude	0.014	0.010	–0.053	0.109***	0.080*	–															
7. Extraversion	0.188***	–0.035	0.200***	0.028	−0.118***	−0.366***	–														
8. Agreeableness	0.256***	–0.027	0.226***	0.029	−0.101**	−0.218***	0.228***	–													
9. Conscientiousness	0.287***	0.129***	0.285***	0.025	−0.127***	0.007	0.092**	0.136***	–												
10. Neuroticism	−0.198***	0.268***	−0.182***	–0.051	0.036	0.085**	−0.188***	−0.160***	–0.019	–											
11. Openness	0.127***	−0.116***	0.026	0.064*	0.026	0.080*	0.104**	0.083*	0.039	−0.089**	–										
12. Emotional privation	−0.101**	–0.020	−0.144***	0.012	0.097**	0.232***	−0.253***	−0.234***	−0.216***	0.263***	–0.037	–									
13. Companionship	–0.062	−0.076*	−0.082*	0.036	0.057	0.226***	−0.221***	−0.155***	−0.156***	0.183***	–0.043	0.775***	–								
14. Stress	−0.073*	0.145***	−0.113***	0.002	0.062	–0.022	–0.036	−0.072*	–0.029	0.465***	–0.032	0.250***	0.125***	–							
15. Job satisfaction	0.160***	–0.039	0.168***	0.115***	–0.045	0.157***	0.124***	0.113***	0.308***	−0.176***	0.137***	−0.315***	−0.228***	−0.236***	–						
16. Vigor	0.210***	–0.011	0.204***	0.004	−0.149***	–0.054	0.220***	0.184***	0.392***	−0.233***	0.091**	−0.455***	−0.360***	−0.200***	0.530***	–					
17. Absorption	0.186***	0.008	0.153***	0.031	−0.075*	–0.017	0.156***	0.177***	0.357***	−0.089**	0.095**	−0.309***	−0.239***	0.012	0.372***	0.671***	–				
18. Dedication	0.122***	–0.042	0.120***	0.028	−0.097**	−0.067*	0.179***	0.163***	0.220***	−0.198***	0.125***	−0.383***	−0.318***	−0.112***	0.389***	0.699***	0.615***	–			
19. Subjective creativity	0.188***	−0.239***	0.133***	0.131***	0.007	0.013	0.258***	0.144**	0.133***	−0.314**	0.423***	−0.179***	−0.186***	−0.119***	0.299***	0.337***	0.293***	0.334***	–		
20. Fluency	0.138***	–0.022	0.059	0.084*	0.014	–0.022	0.066*	0.073*	0.026	–0.034	0.130***	–0.041	–0.049	0.034	0.017	0.080*	0.054	0.079*	0.180***	–	
21. Originality	0.098**	–0.058	0.060	0.037	–0.013	0.017	0.128***	0.070*	–0.019	−0.09**	0.087**	0.018	0.015	0.014	−0.079*	0.055	0.049	0.075*	0.157***	0.506***	–
*M*	35.4	–	–	1.98	3.88	6.69	3.24	3.55	4.06	3.20	3.52	2.68	2.71	2.62	6.98	4.91	5.12	5.04	6.53	3.65	1.73
SD	11.5	–	–	0.95	1.38	2.76	1.17	0.86	0.84	1.17	0.93	1.20	1.13	1.20	2.22	1.06	0.98	1.19	2.46	2.51	0.85
Min value	18	0	0	1	1	0	1	1	1	1	1	1	1	1	1	1	1	1	1	0	0
Max value	70	1	1	4	5	12	5	5	5	5	5	7	7	5	10	7	7	7	10	14	5
Skewness	–	–	–	0.57	–0.95	0.06	–0.11	–0.29	–0.73	–0.179	–0.21	0.90	0.89	0.22	–0.57	–0.50	–0.44	–0.58	–0.35	0.73	–0.09
Kurtosis	–	–	–	–0.71	–0.289	–0.69	–1.05	–0.44	–0.048	–0.995	–0.57	0.63	0.73	–0.88	–0.21	0.34	0.36	0.46	–0.66	0.69	0.50

*N = 922–946; gender was coded as a dummy variable (0 = male, 1 = female); with children was coded as a dummy variable (0 = not, 1 = live with a children)*

***p < 0.001, **p < 0.01, *p < 0.05. WFH, work-from-home.

### Multiple Regression Analyses

Multiple linear regressions, controlling gender, age, number of children, WFH experience and WFH intensity, were calculated in order to assess to what extent preference for solitude and related personality variables could predict negative indicators of well-being (i.e., loneliness at work and perceived stress), positive indicators of well-being (i.e., job satisfaction and dimensions of work engagement) and creative performance (i.e., ‘subjective’ and ‘objective’ creativity). The results of these analyses are summarized in Tables 2–4.

**TABLE 2:** Hierarchical multiple regression analyses for measures of ill-being.

95% Confidence Interval							
**Predictor**	**Estimate**	**SE**	***t***	***p***	**Stand. estimate**	**Lower**	**Upper**
**Emotional privation**							
Intercept	3.43691	0.35024	9.813	<0.001			
Age	0.00863	0.00461	1.873	0.061	0.08247	–0.00392	0.16885
Gender	–0.18433	0.08783	–2.099	0.036	–0.06635	–0.12840	–0.00430
Children	–0.12102	0.10357	–1.168	0.243	–0.05023	–0.13459	0.03414
WFH experience	0.00568	0.03941	0.144	0.885	0.00451	–0.05688	0.06590
WFH intensity	0.02151	0.02714	0.793	0.428	0.02482	–0.03664	0.08628
Preference for solitude	0.05804	0.01435	4.043	<0.001	0.13412	0.06902	0.19922
Extraversion	–0.12378	0.03447	–3.591	<0.001	–0.12070	–0.18666	–0.05473
Agreeableness	–0.17588	0.04455	–3.948	<0.001	–0.12680	–0.18983	–0.06377
Conscientiousness	–0.25088	0.04500	–5.575	<0.001	–0.17834	–0.24112	–0.11557
Neuroticism	0.23568	0.03279	7.188	<0.001	0.22993	0.16715	0.29271
Openness	–0.01511	0.03988	–0.379	0.705	–0.01167	–0.07210	0.04877
**Social companionship**							
Intercept	3.29398	0.34620	9.5147	<0.001			
Age	0.00351	0.00455	0.7719	0.440	0.03525	–0.0544	0.12487
Gender	–0.28815	0.08682	–3.3191	<0.001	–0.10886	–0.1732	–0.04449
Children	–0.00545	0.10238	–0.0532	0.958	–0.00237	–0.0899	0.08515
WFH experience	0.03461	0.03896	0.8884	0.375	0.02883	–0.0349	0.09252
WFH intensity	–0.00673	0.02682	–0.2509	0.802	–0.00815	–0.0719	0.05561
Preference for solitude	0.06537	0.01419	4.6070	<0.001	0.15853	0.0910	0.22606
Extraversion	–0.11255	0.03407	–3.3036	<0.001	–0.11518	–0.1836	–0.04676
Agreeableness	–0.08232	0.04404	–1.8695	0.062	–0.06229	–0.1277	0.00310
Conscientiousness	–0.17018	0.04448	–3.8263	<0.001	–0.12697	–0.1921	–0.06184
Neuroticism	0.16961	0.03241	5.2331	<0.001	0.17366	0.1085	0.23879
Openness	–0.04315	0.03942	–1.0946	0.274	–0.03497	–0.0977	0.02773
**Stress**							
Intercept	0.81603	0.34143	2.390	0.017			
Age	0.00903	0.00449	2.010	0.045	0.0863	0.00206	0.17055
Gender	0.08159	0.08562	0.953	0.341	0.0294	–0.03113	0.08989
Children	–0.19219	0.10097	–1.903	0.057	–0.0798	–0.16206	0.00248
WFH experience	0.02218	0.03842	0.577	0.564	0.0176	–0.04226	0.07748
WFH intensity	0.04262	0.02645	1.611	0.108	0.0492	–0.01074	0.10913
Preference for solitude	–0.03309	0.01399	–2.365	0.018	–0.0765	–0.13998	–0.01301
Extraversion	0.03339	0.03360	0.994	0.321	0.0326	–0.03175	0.09689
Agreeableness	–0.02433	0.04343	–0.560	0.575	–0.0175	–0.07902	0.04392
Conscientiousness	–0.02563	0.04387	–0.584	0.559	–0.0182	–0.07944	0.04300
Neuroticism	0.48389	0.03196	15.139	<0.001	0.4723	0.41104	0.53348
Openness	0.01783	0.03887	0.459	0.647	0.0138	–0.04516	0.07271

*Gender was coded as a dummy variable (0 = male, 1 = female); with children was coded as a dummy variable (0 = not, 1 = live with a children). WFH, work-from-home.*

**TABLE 3:** Hierarchical multiple regression analyses for measures of well-being.

95% Confidence Interval							
**Predictor**	**Estimate**	**SE**	***t***	***p***	**Stand. estimate**	**Lower**	**Upper**
**Job satisfaction**							
Intercept	1.8676	0.65072	2.870	0.004			
Age	–0.0130	0.00856	–1.524	0.128	–0.0672	–0.1537	0.0194
Gender	–0.1128	0.16318	–0.691	0.490	–0.0219	–0.0841	0.0403
Children	0.3248	0.19243	1.688	0.092	0.0727	–0.0118	0.1572
WFH experience	0.1679	0.07323	2.293	0.022	0.0719	0.0104	0.1334
WFH intensity	–0.0291	0.05042	–0.578	0.563	–0.0181	–0.0797	0.0434
Preference for solitude	0.1741	0.02667	6.529	<0.001	0.2170	0.1517	0.2822
Extraversion	0.2496	0.06404	3.898	<0.001	0.1312	0.0652	0.1973
Agreeableness	0.1618	0.08277	1.955	0.051	0.0629	−2.37*e*−4	0.1261
Conscientiousness	0.7186	0.08360	8.596	<0.001	0.2754	0.2126	0.3383
Neuroticism	–0.2630	0.06092	–4.317	<0.001	–0.1384	–0.2012	–0.0755
Openness	0.1880	0.07409	2.537	0.011	0.0783	0.0177	0.1388
**Vigor**							
Intercept	2.85647	0.30364	9.408	<0.001			
Age	0.00139	0.00399	0.349	0.727	0.01489	–0.06892	0.0987
Gender	0.01429	0.07614	0.188	0.851	0.00576	–0.05444	0.0660
Children	0.04642	0.08979	0.517	0.605	0.02156	–0.06029	0.1034
WFH experience	–0.00437	0.03417	–0.128	0.898	–0.00388	–0.06344	0.0557
WFH intensity	–0.05713	0.02353	–2.428	0.015	–0.07377	–0.13340	–0.0141
Preference for solitude	0.00973	0.01244	0.782	0.434	0.02516	–0.03799	0.0883
Extraversion	0.12183	0.02988	4.077	<0.001	0.13294	0.06895	0.1969
Agreeableness	0.08423	0.03862	2.181	0.029	0.06796	0.00680	0.1291
Conscientiousness	0.43452	0.03901	11.139	<0.001	0.34567	0.28477	0.4066
Neuroticism	–0.16056	0.02843	–5.649	<0.001	–0.17530	–0.23621	–0.1144
Openness	0.05215	0.03457	1.509	0.132	0.04507	–0.01356	0.1037
**Absorption**							
Intercept	2.64817	0.29259	9.0507	<0.001			
Age	0.00568	0.00385	1.4768	0.140	0.06623	–0.0218	0.1542
Gender	–0.00446	0.07337	–0.0608	0.951	–0.00196	–0.0652	0.0613
Children	–0.05653	0.08653	–0.6533	0.514	–0.02861	–0.1146	0.0573
WFH experience	0.01261	0.03293	0.3830	0.702	0.01221	–0.0503	0.0748
WFH intensity	–0.01219	0.02267	–0.5376	0.591	–0.01715	–0.0798	0.0455
Preference for solitude	0.01322	0.01199	1.1023	0.271	0.03725	–0.0291	0.1036
Extraversion	0.08186	0.02879	2.8430	0.005	0.09735	0.0301	0.1646
Agreeableness	0.10788	0.03722	2.8988	0.004	0.09486	0.0306	0.1591
Conscientiousness	0.36664	0.03759	9.7535	<0.001	0.31787	0.2539	0.3818
Neuroticism	–0.03005	0.02739	–1.0969	0.273	–0.03575	–0.0997	0.0282
Openness	0.05353	0.03331	1.6068	0.108	0.05041	–0.0112	0.1120
**Dedication**							
Intercept	3.54799	0.36573	9.7011	<0.001			
Age	–0.00247	0.00481	–0.5142	0.607	–0.02365	–0.11394	0.06663
Gender	–0.05503	0.09171	–0.6001	0.549	–0.01983	–0.08467	0.04502
Children	0.02415	0.10815	0.2233	0.823	0.01003	–0.07814	0.09820
WFH experience	0.02975	0.04116	0.7227	0.470	0.02363	–0.04053	0.08779
WFH intensity	–0.05043	0.02834	–1.7797	0.075	–0.05824	–0.12247	0.00598
Preference for solitude	–0.00132	0.01499	–0.0882	0.930	–0.00306	–0.07109	0.06497
Extraversion	0.10181	0.03599	2.8287	0.005	0.09935	0.03042	0.16829
Agreeableness	0.10131	0.04652	2.1779	0.030	0.07310	0.00723	0.13897
Conscientiousness	0.27038	0.04699	5.7544	<0.001	0.19236	0.12676	0.25797
Neuroticism	–0.14998	0.03424	–4.3805	<0.001	–0.14644	–0.21205	–0.08083
Openness	0.12516	0.04164	3.0058	0.003	0.09673	0.03357	0.15989

*Gender was coded as a dummy variable (0 = male, 1 = female); with children was coded as a dummy variable (0 = not, 1 = live with a children). WFH, work-from-home.*

**TABLE 4:** Hierarchical multiple regression analyses for measures of creative performance.

95% Confidence Interval							
**Predictor**	**Estimate**	**SE**	***t***	***p***	**Stand. estimate**	**Lower**	**Upper**
**Subjective creativity**							
Intercept	2.14904	0.35129	6.1176	<0.001			
Age	4.75*e*−4	0.00462	0.1029	0.918	0.0863	0.00206	0.17055
Gender	–0.44003	0.08809	–4.9951	<0.001	0.0294	–0.03113	0.08989
Children	–0.01587	0.10388	–0.1528	0.879	–0.0798	–0.16206	0.00248
WFH experience	0.09942	0.03953	2.5150	0.012	0.0176	–0.04226	0.07748
WFH intensity	–0.00223	0.02722	–0.0820	0.935	0.0492	–0.01074	0.10913
Preference for solitude	0.03185	0.01440	2.2121	0.027	–0.0765	–0.13998	–0.01301
Extraversion	0.20867	0.03457	6.0359	<0.001	0.0326	–0.03175	0.09689
Agreeableness	0.04753	0.04468	1.0638	0.288	–0.0175	–0.07902	0.04392
Conscientiousness	0.16840	0.04513	3.7313	<0.001	–0.0182	–0.07944	0.04300
Neuroticism	–0.22924	0.03289	–6.9707	<0.001	0.4723	0.41104	0.53348
Openness	0.49183	0.04000	12.296	<0.001	0.0138	–0.04516	0.07271
**Fluency**							
Intercept	0.8832	0.7984	1.106	0.269			
Age	0.0390	0.0105	3.715	<0.001	0.17811	0.08401	0.27222
Gender	0.0382	0.2002	0.191	0.849	0.00657	–0.06102	0.07416
Children	–0.4237	0.2361	–1.795	0.073	–0.08403	–0.17593	0.00787
WFH experience	0.1658	0.0899	1.846	0.065	0.06289	–0.00399	0.12976
WFH intensity	0.0140	0.0619	0.226	0.821	0.00771	–0.05924	0.07465
Preference for solitude	–0.0246	0.0327	–0.753	0.452	–0.02719	–0.09810	0.04372
Extraversion	0.0435	0.0786	0.554	0.580	0.02028	–0.05157	0.09213
Agreeableness	0.0805	0.1016	0.793	0.428	0.02773	–0.04093	0.09639
Conscientiousness	–0.0305	0.1026	–0.298	0.766	–0.01037	–0.07875	0.05801
Neuroticism	0.0198	0.0747	0.265	0.791	0.00922	–0.05917	0.07760
Openness	0.2807	0.0909	3.088	0.002	0.10357	0.03774	0.16940
**Originality**							
Intercept	1.24137	0.26762	4.638	<0.001			
Age	0.00703	0.00352	1.997	0.046	0.09603	0.00166	0.1904
Gender	–0.04410	0.06711	–0.657	0.511	–0.02270	–0.09047	0.0451
Children	–0.06287	0.07914	–0.794	0.427	–0.03730	–0.12945	0.0549
WFH experience	0.01957	0.03012	0.650	0.516	0.02220	–0.04486	0.0893
WFH intensity	–0.00567	0.02074	–0.273	0.785	–0.00935	–0.07648	0.0578
Preference for solitude	0.01108	0.01097	1.010	0.313	0.03659	–0.03451	0.1077
Extraversion	0.08194	0.02634	3.111	0.002	0.11421	0.04216	0.1863
Agreeableness	0.03480	0.03404	1.022	0.307	0.03587	–0.03298	0.1047
Conscientiousness	–0.04921	0.03438	–1.431	0.153	–0.05001	–0.11858	0.0186
Neuroticism	–0.03577	0.02505	–1.428	0.154	–0.04989	–0.11847	0.0187
Openness	0.04003	0.03047	1.314	0.189	0.04419	–0.02182	0.1102

*Gender was coded as a dummy variable (0 = male, 1 = female); with children was coded as a dummy variable (0 = not, 1 = live with a children). WFH, work-from-home.*

For the sub-dimension of loneliness at work related to emotional deprivation (Table 2), the results yielded an overall significant effect, *F*(11,910) = 19.81, *p* < 0.001, *R*^2^= 0.19, with a significant contribution of gender, preference for solitude and personality variables. Indeed, male teleworkers tended to report more loneliness at work than female teleworkers (β = −0.18). Moreover, emotional deprivation was higher among individuals with a higher preference for solitude (β = 0.06) and with neuroticism traits (β = 0.24). In contrast, emotional deprivation was negatively related to individuals with higher levels of extraversion (β = −0.12), agreeableness (β = −0.18) and conscientiousness (β = −0.25). For the lack of social companionship, the results yielded an overall significant effect, *F*(11,910) = 12.55, *p* < 0.001, *R*^2^= 0.13, with a significant contribution of gender (β = −0.29). Moreover, lack of social companionship was higher among individuals with a higher preference for solitude (β = 0.07) and with neuroticism traits (β = 0.17). In contrast, lack of social companionship was negatively related to individuals with higher levels of extraversion (β = −0.11) and conscientiousness (β = −0.17). For the symptoms of stress, the results yielded an overall significant effect, *F*(11,910) = 25.09, *p* < 0.001, *R*^2^= 0.22, with a major contribution of neuroticism traits (β = 0.48), indicating that individuals with high levels of neuroticism expressed more symptoms of stress. Moreover, stress was lower among individuals with a higher preference for solitude (β = −0.03) and among older employees (β = 0.009).

For job satisfaction (Table 3), the results yielded an overall significant effect, *F*(11,910) = 19.45, *p* < 0.001, *R*^2^= 0.19. Among control variables, only WFH experience was positively associated with job satisfaction (β = 0.17). As expected, preference for solitude was positively related to job satisfaction (β = 0.17) and also extraversion (β = 0.25), agreeableness (β = 0.16), conscientiousness (β = 0.72) and openness (β = 0.19), whereas neuroticism was negatively associated with job satisfaction (β = −0.26). For the components of work engagement, the results showed an overall significant effect, *F*(11,910) = 26.20, *p* < 0.001, *R*^2^= 0.24 for vigor, *F*(11,910) = 16.05, *p* < 0.001, *R*^2^= 0.16 for absorption, and *F*(11,910) = 11.16, *p* < 0.001, *R*^2^= 0.12 for dedication. Among control variables, only WFH intensity was negatively associated with the sub-dimension of vigor at work (β = −0.06). As expected, personality variables related to the Big Five traits were associated with sub-dimensions of work engagement, but not the preference for solitude.

For the “subjective” measure of creativity (Table 4), the results yielded an overall significant effect, *F*(11,910) = 39.20, *p* < 0.001, *R*^2^= 0.32. The gender variable explained perceived creativity when entering the preference for solitude and personality variables in the regression. Males perceived themselves more creative than females (β = −0.44). WFH experience was also positively associated with perceived creativity (β = 0.10). Preference for solitude and personality variables were associated with perceived creativity.

Finally, concerning the “objective” measures of creativity (Table 4), as expected openness was positively associated with the number of ideas produced (β = 0.28), and extraversion was positively associated with the originality of ideas produced (β = 0.08). The other personality characteristics were not related to fluency or originality scores. Moreover, age was positively associated with the number of ideas produced (β = 0.04), and weakly associated with their originality (β = 0.007).

As expected in our first hypothesis, the experience or intensity of telework alone did not explain the effects of WFH, variables referring to the preference for solitude or Big Five traits often appeared to be related to loneliness at work, stress, job satisfaction, work engagement and creative performance. To explore the data thoroughly, we used a person-centered approach to identify the psychological profiles of employees which fit WFH.

### Cluster Analysis

**FIGURE 1:**
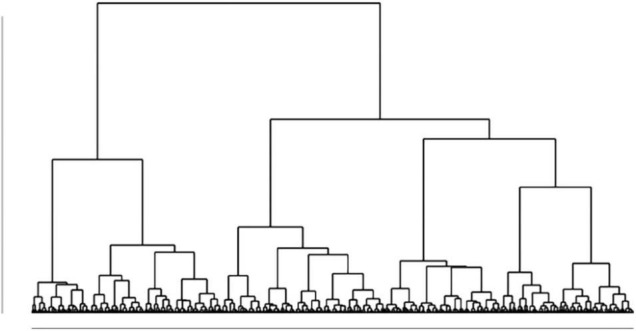
The dendrogram.

**FIGURE 2:**
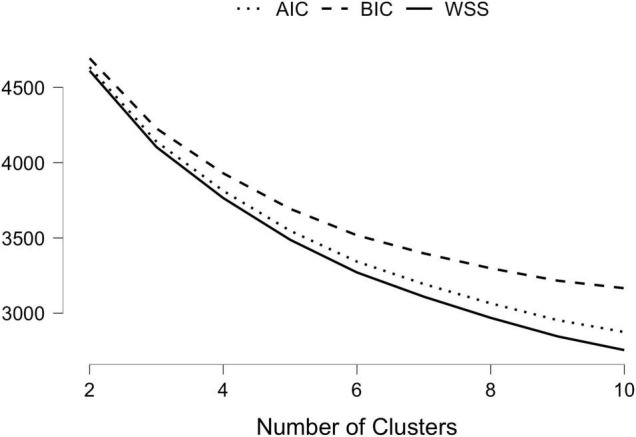
The elbow method plot.

**FIGURE 3:**
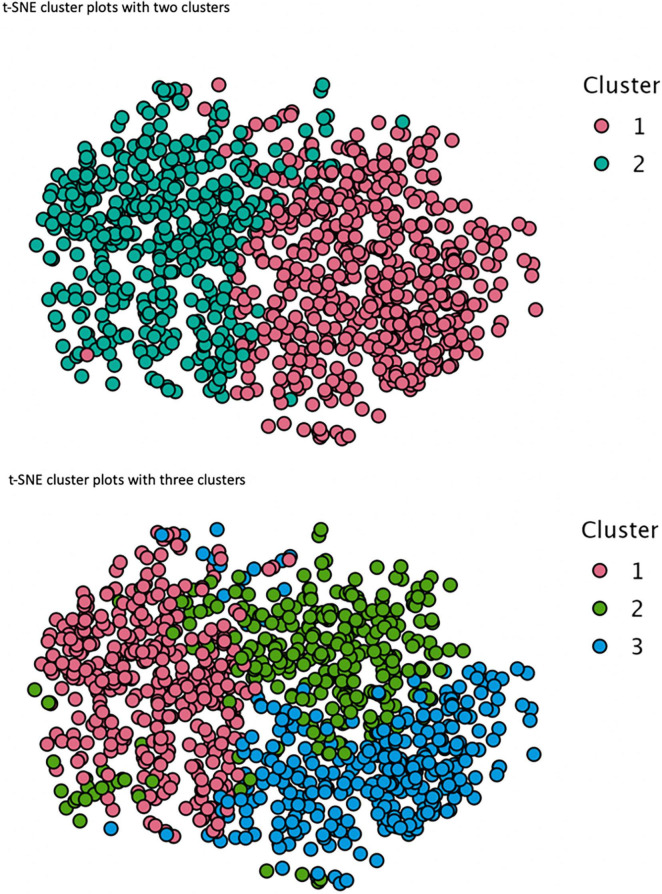
t-SNE cluster plots for two and three clusters.

Hierarchical clustering analysis (Ward’s method) and *k*-means non-hierarchical clustering analysis were performed on the five standardized scores for the preference for solitude variable and personality scores (i.e., extraversion, agreeableness, conscientiousness, neuroticism and openness). First, the results showed that the best number of clusters was two. We then compared different solutions (*k* = 2 and *k* = 3) to form clusters with *k*-means analysis. The three-profile subgroup model fitted the data slightly better (AIC = 4140.27, BIC = 4227.61) than the two-profile subgroup model (AIC = 4635.32, BIC = 4693.54) considering the smaller fit indices. However, the increment in model fit of the three-group model from the two-group model was not significant. Complementary visual techniques based on the inspection of the dendrogram (Figure 1), elbow method plot (Figure 2), and t-SNE cluster plots (Figure 3) confirmed that the solution with two clusters was better than the solution with three less-distinct clusters. This analysis is not consistent with a prior study in which three profiles were identified among a wider population than employees working from home (Michinov and Michinov, 2021).

**FIGURE 4:**
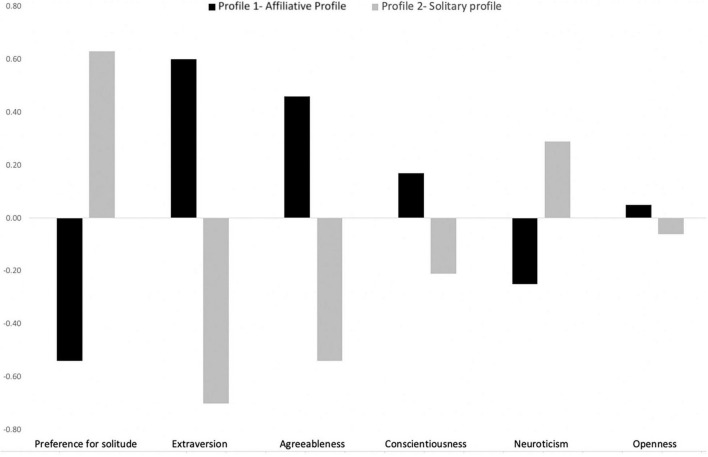
Two profiles based on personality variables.

Figure 4 describes the two profiles retained in the present study. Profile 1, named “Affiliative,” was represented by low levels of preference for solitude and neuroticism, high levels of extraversion and agreeableness, and moderate levels of conscientiousness and openness. This profile concerned 53.91% of the sample (*N* = 510). Profile 2 was labeled “Solitary” and was characterized by high levels of preference for solitude and neuroticism, low levels of extraversion and agreeableness, and moderate levels of conscientiousness and openness. It concerned 46.09% of the sample (*N* = 436). A chi-square analysis showed that gender was equally distributed across the two profiles, χ^2^(1, *N* = 946) = 1.18, *p* = 0.276.

To see whether the two clusters identified were distinct and whether each variable contributed significantly to cluster formation, a MANOVA was conducted with cluster membership as the independent variable and the five personality variables as dependent variables. The overall model was significant, revealing that each personality variable appeared to vary significantly between the two profiles [Wilks’ Λ = 0.32, *F*(6,939) = 328, *p* < 0.001]. The two profiles differ significantly on the preference for solitude and the Big Five personality variables, except on openness with a marginal difference between the two profiles. The details of means in each cluster are shown in Table 5.

**TABLE 5:** Mean differences and standard deviations (SD) in study variables among the two profiles.

	“Affiliative” profile Mean (SD)	“Solitary” profile Mean (SD)	*t*	*p*	Cohen’s *d*
***Personality variables***					
Preference for solitude	5.20^a^ (2.19)	8.43^b^ (2.31)	-22.0	<0.001	–1.43
Extraversion	3.94^a^ (0.85)	2.42^b^ (0.93)	26.2	<0.001	1.71
Agreeableness	3.95^a^ (0.71)	3.09^b^ (0.79)	17.7	<0.001	1.15
Conscientiousness	4.21^a^ (0.82)	3.89^a^ (0.85)	5.95	<0.001	0.39
Neuroticism	2.91^a^ (1.11)	3.54^b^ (1.14)	–8.62	<0.001	–0.56
Openness	3.56^a^ (0.95)	3.46^a^ (0.90)	1.64	<0.10	0.11
***Outcome variables***					
Emotional deprivation	2.33^a^ (1.02)	3.09^b^ (1.26)	-10.3	<0.001	–0.67
Social companionship	2.41^a^ (0.95)	3.06^b^ (1.23)	–9.18	<0.001	–0.60
Stress	2.54^a^(1.18)	2.73^b^ (1.21)	–2.44	0.015	–0.16
Job satisfaction	7.18^a^ (2.12)	6.73^b^ (2.31)	3.14	0.002	0.21
Vigor	5.14^a^ (0.99)	4.64^b^ (1.08)	7.28	<0.001	0.48
Absorption	5.25^a^ (0.93)	4.96^b^ (1.00)	4.51	<0.001	0.29
Dedication	5.23^a^ (1.15)	4.81^b^ (1.21)	5.57	<0.001	0.36
Subj. creativity	4.99^a^ (1.24)	4.48^b^ (1.34)	6.10	<0.001	0.40
Fluency	3.85^a^ (2.58)	3.42^b^ (2.41)	2.61	0.009	0.17
Originality	1.79^a^ (0.83)	1.66^b^ (0.86)	1.74	0.016	0.16

*N = 510 for “Affiliative” profile, N = 436 for “Solitary” profile. Different superscripts in a row indicate significant differences.*

Then, in order to identify the profile of teleworkers at risk, ANOVAs were conducted with cluster membership as the independent variable and well-being and creativity measures as dependent variables. With regard to well-being measures, results showed significant differences between the two clusters (see Table 5). *Post-hoc* tests showed that the individuals with a “Solitary” profile reported more loneliness at work (i.e., emotional deprivation and social companionship), higher levels of stress, and lower levels of job satisfaction and work engagement (i.e., vigor, absorption and dedication) than the individuals with an “Affiliative” profile. As regards the effects of profiles on creativity measures, results showed that individuals with a “Solitary” profile perceived themselves as less creative and actually produced fewer ideas (fluency score) than those with an “Affiliative” profile. For the originality of ideas, the difference was also significant between the two profiles indicating that according to the assessment of external coders the ideas produced were more original among individuals with an “Affiliative” than a “Solitary” profile.

Overall, these results confirm that the psychological profiles related to a preference for solitude and personality variables associated with the Big Five traits influence employees’ well-being and creativity performance during WFH.

## Discussion

With the COVID-19 pandemic, governments implemented successive lockdowns that forced employees to WFH. This crisis raises the question of the effects of mandatory WFH on employees’ well-being and performance, and whether these effects are the same for all teleworkers. As the review of the literature suggested (Allen et al., 2015; Anderson et al., 2015; Götz et al., 2021; Langvik et al., 2021; Liu et al., 2021; Michinov and Michinov, 2021; Volk et al., 2021; Wang et al., 2021), we expected that teleworkers’ well-being and creativity during WFH would depend on their psychological profile.

In accordance with our first hypothesis, the present research provides empirical evidence on the relationships between preference for solitude, Big Five traits, and indicators of well-being and creativity during WFH. Indeed, the analyses based on multiple regression analyses revealed that the effects of preference for solitude and some personality traits related to the Big Five dimensions were more predictive of loneliness at work, stress, job satisfaction, work engagement or creative performance than demographic factors or the experience or intensity of WFH.

The cluster analysis based on the preference for solitude variable and personality factors (extraversion, agreeableness, conscientiousness, neuroticism and openness) showed a significant effect of psychological profiles on well-being at work and creativity, and extend some previous findings by showing profiles of employees at risk when teleworking (e.g., Anderson et al., 2015; Wilmot et al., 2019; Götz et al., 2021; Langvik et al., 2021; Liu et al., 2021; Michinov and Michinov, 2021; Volk et al., 2021). Contrary to our second hypothesis, the present results did not reveal exactly the same psychological profiles as those observed in the general population during the first lockdown in France (Michinov and Michinov, 2021). Indeed, we did not find three profiles but two profiles of teleworkers (named “affiliative” and “solitary”). We explain this difference in results by two main reasons. First, our study is based on a specific sample of teleworkers and at a different period of the COVID-19 pandemic (i.e., the second lockdown in France with less strict restrictions, and the experience of the population of the first lockdown). Second, the scale used to measure the Big Five traits is not the same as the one used in Michinov and Michinov (2021). Thus, the present study takes into account in the profiles the variable of preference for solitude and the five personality traits and not only three personality traits as in the study by Michinov and Michinov (2021). The identification of two psychological profiles (“affiliative” and “solitary”) in our study with opposing patterns in neuroticism, extraversion, conscientiousness, agreeableness and preference for solitude may enable significantly different experiences during WFH to be predicted. The combined effects of high levels of extraversion, conscientiousness and agreeableness, and low levels of neuroticism and preference for solitude (“affiliative” profile) were found to be related to lower loneliness at work, lower stress and higher job satisfaction than the combined effects of high neuroticism, high preference for solitude and low extraversion (“solitary” profile). These results suggested that a combination of traits characterizing a resilient profile (“affiliative”) seems to help a person cope with social isolation and stress during WFH and achieve higher job satisfaction and work engagement, while the over-sensitive profile (“solitary”) may be interpreted as reinforcing the perception of the negative aspects of WFH.

These results provide a contribution to the literature on the effects of personality traits on employees’ acceptability of telework (O’Neill et al., 2009; Allen et al., 2015; Anderson et al., 2015; Wang et al., 2021), in particular mandatory WFH (Kniffin et al., 2021) on well-being and creativity, confirming that not all remote workers are similar and should be considered differently (Donati et al., 2021; Pathak et al., 2021). More specifically, this study provides empirical evidence from a large sample of employees that extraversion and low preference for solitude potentially protect against potential deleterious effects of mandatory WFH, and that neuroticism is a risk factor. Extraverted individuals seem to consider WFH interesting which is somehow contradictory in the sense that much research emphasizes their suitability for social environments and working in a group to reach their full potential (O’Neill et al., 2014a). However, one could consider that their present high level of work satisfaction could be due to the digital solutions and, more specifically extensive use of videoconferencing systems for social interaction that have been enhanced during the period of WFH, enabling extraverts to continue to reach out with their ideas and communicative skills. Indeed, some recent studies during the COVID-19 pandemic have shown that extraversion is related to more active seeking of social support and adaptative coping strategies (Volk et al., 2021). By contrast, individuals with a “solitary” profile (i.e., introverted individuals with a preference for solitude and a high level of neuroticism) may experience more isolation from the work team because they are less able to maintain contact with their colleagues, and less likely to capitalize on positive emotions (Wilmot et al., 2019). In future studies, we need to investigate further the social contacts and communication established during WFH by individuals with “affiliative” and “solitary” profiles.

The major contribution of this study lies in the identification of risk profiles of teleworkers for occupational health and well-being, but also regarding employees’ creativity. To the best of our knowledge, the present results on objective measures of creativity during mandatory WFH are original, as very few studies have focused on creativity during teleworking (Vega et al., 2015; Mercier et al., 2021). Our results show that high levels of extraversion, consciousness, agreeableness and low levels of neuroticism and preference for solitude are related to higher scores in a divergent creative thinking task, whereas introverted and neurotic individuals have lower scores (number and originality of ideas produced). Thus, telecommuting is therefore not conducive to creativity for all employees, contrary to the findings of the study by Vega et al. (2015). Future research is necessary to replicate these results on other tasks and to measure the underlying psychological processes (e.g., emotional regulation, concentration and attention focus).

### Limitations and Perspectives

The large sample of teleworkers from various organizations, the use of validated measures of ill- and well-being, and the inclusion of ‘objective’ indicators of creativity represent strengths of the present study. Although our research has revealed insights into the effects of distinct psychological profiles of teleworkers on well-being and ‘objective’ performance measures of creativity during WFH, this study has some limitations. First, concerning the indicators of well-being (loneliness at work, stress, job satisfaction and work engagement) the study relied only on self-report measures. Additionally, it is not possible to assess whether well-being and performance actually changed for these people during the lockdowns. Future studies could test whether similar profiles can be replicated at another time and monitor how they evolve over time. Second, the study has limitations that relate to the individual differences and personality variables measured. In the present research, we examined the effects of individual differences in tolerance to social isolation and preference for solitude. Other individual differences may influence telework attitudes such as procrastination, self-discipline or segmentation preferences (Kreiner, 2006; Allen et al., 2021; Kerman et al., 2021). For example, segmentation preferences refer to the degree to which an employee prefers to keep work- and private-life separate (Allen et al., 2021). A relationship was found between the employees’ segmentation preferences and work-related conflict, indicating that ‘segmentors’ could be able to manage their work-life balance more effectively during mandatory WFH in the context of the COVID-19 pandemic (Allen et al., 2021). Finally, the study is based on a large sample of French teleworkers, and future studies could investigate a more diversified population to generalize the findings in different organizational contexts and in other countries. Unfortunately, this study does not include measures of social contact besides colleagues, and it would be relevant to include measures of social activities and the use of videoconferencing systems. Despite these limitations, this study has several implications for organizations and managers who develop telework practices and virtual teams.

### Conclusion and Practical Implications

The COVID-19 pandemic has dramatically changed social interactions and work settings. Virtual teams and WFH have rapidly increased and forced workers to adapt to these new forms of work. The pandemic has revealed some groups of workers who are more vulnerable than others, for example, based on their age, ethnicity, gender or personality. The present study provides evidence of employees’ profiles related to preference for solitude and the Big Five personality traits (“affiliative” and “solitary” profiles), and encourages reflection on the needs of teleworkers. As suggested by Kramer and Kramer (2020, p. 2), WFH seem to “require selection of workers who are better suited to work from home, training of such workers on more efficient methods of remote work, and greater monitoring of the quality and productivity of those assigned to work from home.” First, based on the detected profiles, organizations and managers could try to detect the employees’ profiles and organize telework practices in order to guarantee employees’ performance, creativity and well-being. Knowing the different levels of tolerance and sensitivity to professional isolation could help managers to propose support practices and adapted communication tools (e.g., formal and informal networks). The support tools during remote working would also be more useful for inexperienced teleworkers or employees who are uncomfortable with digital technology. Employees should be informed of the effects of remote work, and some guidelines adapted to their personality profile would be provided to arrange their telecommuting time. Experienced teleworkers could also provide informational and social support *via* communication tools or private rooms on telecommuting platforms. Managers must continue to allow employees flexibility in managing telework according to their personality profiles, their working conditions at home (presence of the spouse, children, etc.) and feedback on their remote work experiences. Managers’ consideration of employee profiles should help to avoid deepening the inequalities at work caused by digitalization and new forms of work organization. Of course, although the remote working experience may be influenced by psychological factors, companies have to adopt organizational strategies reflecting their own situation. In the future, practitioners need to reflect on working outside the office in different places, and this organization of where people work should take into account the characteristics of the work and employee preferences. Human resources managers should not forget that the effects of WFH could differ according to the profile of employees. Future research is also needed to improve understanding of what determines employees’ preferences about workplaces.

## Data Availability Statement

The datasets generated and/or analyzed during the current study are available from: https://doi.org/10.17605/OSF.IO/DKRWY.

## Ethics Statement

The studies involving human participants were reviewed and approved by Ethical approval was granted by the Ethics Committee of the University of Rennes 2 (N° 2021-030). The patients/participants provided their written informed consent to participate in this study.

## Author Contributions

EM and NM contributed to the material preparation and analyses. EM wrote the first draft of the manuscript. All authors commented on previous versions of the manuscript and contributed to the study conception and design and data collection. All authors read and approved the final manuscript.

## Conflict of Interest

The authors declare that the research was conducted in the absence of any commercial or financial relationships that could be construed as a potential conflict of interest.

## Publisher’s Note

All claims expressed in this article are solely those of the authors and do not necessarily represent those of their affiliated organizations, or those of the publisher, the editors and the reviewers. Any product that may be evaluated in this article, or claim that may be made by its manufacturer, is not guaranteed or endorsed by the publisher.

## References

[B1] Afonso P. Fonseca M. Teodoro T. (2021). Evaluation of anxiety, depression and sleep quality in full-time teleworkers. *J. Public Health* 10.1093/pubmed/fdab164 [Epub ahead of print]. 34036369 PMC8202819

[B2] Ajzen M. Taskin L. (2021). The re-regulation of working communities and relationships in the context of flexwork: a spacing identity approach. *Inf. Organ.* 31:100364. 10.1016/j.infoandorg.2021.100364

[B3] Allen T. D. Golden T. D. Shockley K. M. (2015). How effective is telecommuting? Assessing the status of our scientific findings. *Psychol. Sci. Public Interest* 16 40–68. 10.1177/1529100615593273 26403188

[B4] Allen T. D. Merlo K. Lawrence R. C. Slutsky J. Gray C. E. (2021). Boundary management and work-nonwork balance while working from home. *Appl. Psychol.* 70 60–84. 10.1111/apps.12300

[B5] Anderson A. J. Kaplan S. A. Vega R. P. (2015). The impact of telework on emotional experience: when, and for whom, does telework improve daily affective well-being? *Eur. J. Work Organ. Psychol.* 24 882–897. 10.1080/1359432X.2014.966086

[B6] Baumeister R. F. Leary M. R. (1995). The need to belong: desire for interpersonal attachments as a fundamental human motivation. *Psychol. Bull.* 117 497–529. 10.1037/0033-2909.117.3.497 7777651

[B7] Biron M. van Veldhoven M. (2016). When control becomes a liability rather than an asset: comparing home and office days among part-time teleworkers: within-individual Study on Part-time Telework. *J. Organ. Behav.* 37 1317–1337. 10.1002/job.2106

[B8] Boell S. K. Cecez-Kecmanovic D. Campbell J. (2016). Telework paradoxes and practices: the importance of the nature of work. *New Technol. Work Employ.* 31 114–131. 10.1111/ntwe.12063

[B9] Burger J. M. (1995). Individual differences in preference for solitude. *J. Res. Personal.* 29 85–108. 10.1006/jrpe.1995.1005

[B10] Charalampous M. Grant C. A. Tramontano C. Michailidis E. (2019). Systematically reviewing remote e-workers’ well-being at work: a multidimensional approach. *Eur. J. Work Organ. Psychol.* 28 51–73. 10.1080/1359432X.2018.1541886

[B11] Clark L. A. Karau S. J. Michalisin S. D. (2012). Work from home attitudes and the ‘big five’ personality dimensions. *J. Manag. Policy Pract.* 13 31–46.

[B12] Cohen J. Cohen J.(eds) (2003). *Applied Multiple Regression/Correlation Analysis for the Behavioral Sciences*, 3rd Edn. Mahwah, NJ: L. Erlbaum Associates.

[B13] Courtois R. Petot J.-M. Plaisant O. Allibe B. Lignier B. Réveillère C. (2020). Validation française du big five inventory à 10 items (bfi-10). *L’Encéphale* 46 455–462. 10.1016/j.encep.2020.02.006 32331765

[B14] de Macêdo T. A. M. Cabral E. L. dos S. Silva Castro W. R. de Souza Junior C. C. da Costa Junior J. F. (2020). Ergonomics and telework: a systematic review. *Work* 66 777–788. 10.3233/WOR-203224 32925139

[B15] Delanoeije J. Verbruggen M. (2020). Between-person and within-person effects of telework: a quasi-field experiment. *Eur. J. Work Organ. Psychol.* 29 795–808. 10.1080/1359432X.2020.1774557

[B16] Derks D. Bakker A. B. Peters P. van Wingerden P. (2016). Work-related smartphone use, work–family conflict and family role performance: the role of segmentation preference. *Hum. Relat.* 69 1045–1068. 10.1177/0018726715601890

[B17] Donati S. Viola G. Toscano F. Zappalà S. (2021). Not all remote workers are similar: technology acceptance, remote work beliefs, and wellbeing of remote workers during the second wave of the covid-19 pandemic. *Int. J. Environ. Res. Public. Health* 18:12095. 10.3390/ijerph182212095 34831849 PMC8623028

[B18] Elo A.-L. Leppänen A. Jahkola A. (2003). Validity of a single-item measure of stress symptoms. *Scand. J. Work Environ. Health* 29, 444–451. 10.5271/sjweh.752 14712852

[B19] Elo A. L. Leppänen A. Lindstrom K. Ropponen T. (1992). *Occupational Stress Questionnaire: User’s Instructions.* Helsinski: Institute of Occupational Health Care.

[B20] Entringer T. M. Gosling S. D. (2021). Loneliness during a nationwide lockdown and the moderating effect of extroversion. *Soc. Psychol. Personal. Sci.* 10.1177/19485506211037871 [Epub ahead of print].

[B21] Faul F. Erdfelder E. Lang A.-G. Buchner A. (2007). G*Power 3: a flexible statistical power analysis program for the social, behavioral, and biomedical sciences. *Behav. Res. Methods* 39 175–191. 10.3758/BF03193146 17695343

[B22] Fuller C. M. Simmering M. J. Atinc G. Atinc Y. Babin B. J. (2016). Common methods variance detection in business research. *J. Bus. Res.* 69 3192–3198. 10.1016/j.jbusres.2015.12.008

[B23] Gajendran R. S. Harrison D. A. (2007). The good, the bad, and the unknown about telecommuting: meta-analysis of psychological mediators and individual consequences. *J. Appl. Psychol.* 92 1524–1541. 10.1037/0021-9010.92.6.1524 18020794

[B24] Gajendran R. S. Harrison D. A. Delaney-Klinger K. (2015). Are telecommuters remotely good citizens? Unpacking telecommuting’s effects on performance *via* i-deals and job resources: personnel psychology. *Pers. Psychol.* 68 353–393. 10.1111/peps.12082

[B25] Galanti T. Guidetti G. Mazzei E. Zappalà S. Toscano F. (2021). Work from home during the covid-19 outbreak: the impact on employees’ remote work productivity, engagement and stress. *J. Occup. Environ. Med.* 63 e426-e432. 10.1097/JOM.0000000000002236 33883531 PMC8247534

[B26] Ghislieri C. Dolce V. Sanseverino D. Wodociag S. Vonthron A.-M. Vayre É (2022). Might insecurity and use of ICT enhance internet addiction and exhaust people? A study in two European countries during emergency remote working. *Comput. Hum. Behav.* 126:107010. 10.1016/j.chb.2021.107010 PMC9758255 36569411

[B27] Gillet N. Colombat P. Michinov E. Pronost A.-M. Fouquereau E. (2013). Procedural justice, supervisor autonomy support, work satisfaction, organizational identification and job performance: the mediating role of need satisfaction and perceived organizational support. *J. Adv. Nurs.* 69 2560–2571. 10.1111/jan.12144 23551132

[B28] Glenn Dutcher E. (2012). The effects of telecommuting on productivity: an experimental examination. The role of dull and creative tasks. *J. Econ. Behav. Organ.* 84 355–363. 10.1016/j.jebo.2012.04.009

[B29] Golden T. D. Gajendran R. S. (2019). Unpacking the role of a telecommuter’s job in their performance: examining job complexity, problem solving, interdependence, and social support. *J. Bus. Psychol.* 34 55–69. 10.1007/s10869-018-9530-4

[B30] Golden T. D. Veiga J. F. Dino R. N. (2008). The impact of professional isolation on teleworker job performance and turnover intentions: does time spent teleworking, interacting face-to-face, or having access to communication-enhancing technology matter? *J. Appl. Psychol.* 93 1412–1421. 10.1037/a0012722 19025257

[B31] Götz F. M. Gvirtz A. Galinsky A. D. Jachimowicz J. M. (2021). How personality and policy predict pandemic behavior: understanding sheltering-in-place in 55 countries at the onset of COVID-19. *Am. Psychol.* 76 39–49. 10.1037/amp0000740 33475389

[B32] Graham M. Weale V. Lambert K. A. Kinsman N. Stuckey R. Oakman J. (2021). Working at home: the impacts of covid 19 on health, family-work-life conflict, gender, and parental responsibilities. *J. Occup. Environ. Med.* 63 938–943. 10.1097/JOM.0000000000002337 34325437 PMC8562911

[B33] Grant C. A. Wallace L. M. Spurgeon P. C. Tramontano C. Charalampous M. (2019). Construction and initial validation of the E-Work Life Scale to measure remote e-working. *Empl. Relat.* 41 16–33. 10.1108/ER-09-2017-0229

[B34] Guler M. A. Guler K. Guneser Gulec M. Ozdoglar E. (2021). Working from home during a pandemic: investigation of the impact of covid-19 on employee health and productivity. *J. Occup. Environ. Med.* 63 731–741. 10.1097/JOM.0000000000002277 34091577

[B35] Hair J. F. Black W. C. Babin B. J. Anderson R. E. (2010). *Multivariate Data Analysis: A Global Perspective.* London: Pearson Education.

[B36] Hammer L. B. Neal M. B. Newsom J. T. Brockwood K. J. Colton C. L. (2005). A longitudinal study of the effects of dual-earner couples’ utilization of family-friendly workplace supports on work and family outcomes. *J. Appl. Psychol.* 90 799–810. 10.1037/0021-9010.90.4.799 16060797

[B37] JASP Team (2022). *JASP (Version 0.14.1)[Computer software].* Available online at: https://jasp-stats.org

[B38] Kerman K. Korunka C. Tement S. (2021). Work and home boundary violations during the COVID-19 pandemic: the role of segmentation preferences and unfinished tasks. *Appl. Psychol.* 10.1111/apps.12335 [Epub ahead of print]. 34548734 PMC8444894

[B39] Kniffin K. M. Narayanan J. Anseel F. Antonakis J. Ashford S. P. Bakker A. B. (2021). COVID-19 and the workplace: implications, issues, and insights for future research and action. *Am. Psychol.* 76 63–77. 10.1037/amp0000716 32772537

[B40] Kossek E. E. Lautsch B. A. Eaton S. C. (2006). Telecommuting, control, and boundary management: correlates of policy use and practice, job control, and work–family effectiveness. *J. Vocat. Behav.* 68 347–367. 10.1016/j.jvb.2005.07.002

[B41] Kramer A. Kramer K. Z. (2020). The potential impact of the Covid-19 pandemic on occupational status, work from home, and occupational mobility. *J. Vocat. Behav.* 119:103442. 10.1016/j.jvb.2020.103442 32390661 PMC7205621

[B42] Kreiner G. E. (2006). Consequences of work-home segmentation or integration: a person-environment fit perspective. *J. Organ. Behav.* 27 485–507. 10.1002/job.386

[B43] Langevin V. Boini S. François M. Riou A. (2012). Item unique de mesure des symptômes de stress. *Réf. En Santé Au Trav.* 131 155–156.

[B44] Langvik E. Karlsen H. R. Saksvik-Lehouillier I. Sørengaard T. A. (2021). Police employees working from home during COVID-19 lockdown: those with higher score on extraversion miss their colleagues more and are more likely to socialize with colleagues outside work. *Personal. Individ. Differ.* 179:110924. 10.1016/j.paid.2021.110924 PMC9756409 36540085

[B45] Limesurvey GmbH *LimeSurvey: An Open Source Survey Tool/LimeSurvey GmbH.* Hamburg: Limesurvey GmbH.

[B46] Liu S. Lithopoulos A. Zhang C.-Q. Garcia-Barrera M. A. Rhodes R. E. (2021). Personality and perceived stress during COVID-19 pandemic: testing the mediating role of perceived threat and efficacy. *Personal. Individ. Differ.* 168:110351. 10.1016/j.paid.2020.110351 32863508 PMC7442020

[B47] Masuda A. D. Holtschlag C. Nicklin J. M. (2017). Why the availability of telecommuting matters: the effects of telecommuting on engagement *via* goal pursuit. *Career Dev. Int.* 22 200–219. 10.1108/CDI-05-2016-0064

[B48] Mercier M. Vinchon F. Pichot N. Bonetto E. Bonnardel N. Girandola F. (2021). Covid-19: a boon or a bane for creativity? *Front. Psychol.* 11:601150. 10.3389/fpsyg.2020.601150 33536973 PMC7848087

[B49] Michinov E. Michinov N. (2021). Stay at home! When personality profiles influence mental health and creativity during the COVID-19 lockdown. *Curr. Psychol.* 10.1007/s12144-021-01885-3 [Epub ahead of print]. 34092985 PMC8163587

[B50] Michinov E. Michinov N. Ruiller C. Dodeler V. Chedotel F. (2021). *Dataset About the Project Working-From-Home During Lockdown.* 10.17605/OSF.IO/DKRWY

[B51] Nouri R. Erez M. Lee C. Liang J. Bannister B. D. Chiu W. (2015). Social context: key to understanding culture’s effects on creativity. *J. Organ. Behav.* 36 899–918. 10.1002/job.1923

[B52] Oakman J. Kinsman N. Stuckey R. Graham M. Weale V. (2020). A rapid review of mental and physical health effects of working at home: how do we optimise health? *BMC Public Health* 20:1825. 10.1186/s12889-020-09875-z 33256652 PMC7703513

[B53] O’Neill T. A. Hambley L. A. Bercovich A. (2014b). *Satisfaction and Performance When Working Remotely Measure.* Washington, DC: APA PsycTests. 10.1037/t33762-000

[B54] O’Neill T. A. Hambley L. A. Bercovich A. (2014a). Prediction of cyberslacking when employees are working away from the office. *Comput. Hum. Behav.* 34 291–298. 10.1016/j.chb.2014.02.015

[B55] O’Neill T. A. Hambley L. A. Greidanus N. S. MacDonnell R. Kline T. J. B. (2009). Predicting teleworker success: an exploration of personality, motivational, situational, and job characteristics. *New Technol. Work Employ.* 24 144–162. 10.1111/j.1468-005X.2009.00225.x

[B56] Ozcelik H. Barsade S. G. (2018). No employee an island: workplace loneliness and job performance. *Acad. Manage. J.* 61 2343–2366. 10.5465/amj.2015.1066

[B57] Pathak D. Vijayakumar Bharathi S. Padma Mala E. (2021). The work-life balancing act: a study on the mandatory work from home due to covid-19 on the it and non-it industry sectors. *Int. J. Hum. Cap. Inf. Technol. Prof.* 12, 1–20. 10.4018/IJHCITP.2021070101

[B58] Podsakoff P. M. MacKenzie S. B. Lee J.-Y. Podsakoff N. P. (2003). Common method biases in behavioral research: a critical review of the literature and recommended remedies. *J. Appl. Psychol.* 88 879–903. 10.1037/0021-9010.88.5.879 14516251

[B59] Pulido-Martos M. Cortés-Denia D. Lopez-Zafra E. (2021). Teleworking in times of COVID-19: effects on the acquisition of personal resources. *Front. Psychol.* 12:685275. 10.3389/fpsyg.2021.685275 34248789 PMC8262645

[B60] Rammstedt B. John O. P. (2007). Measuring personality in one minute or less: a 10-item short version of the Big Five Inventory in English and German. *J. Res. Personal.* 41 203–212. 10.1016/j.jrp.2006.02.001

[B61] Ruiller C. Van Der Heijden B. Chedotel F. Dumas M. (2019). “You have got a friend”: the value of perceived proximity for teleworking success in dispersed teams. *Team Perform. Manag. Int. J.* 25 2–29. 10.1108/TPM-11-2017-0069

[B62] Russell E. Daniels K. (2018). Measuring affective well-being at work using short-form scales: implications for affective structures and participant instructions. *Hum. Relat.* 71 1478–1507. 10.1177/0018726717751034 30270934 PMC6146316

[B63] Saris W. E. Gallhofer I. N. (eds) (2014). *Design, Evaluation, and Analysis of Questionnaires for Survey Research: Saris/Design, Evaluation, and Analysis of Questionnaires for Survey Research.* Hoboken, NJ: John Wiley and Sons, 10.1002/9781118634646

[B64] Schaufeli W. B. Bakker A. B. Salanova M. (2006). The measurement of work engagement with a short questionnaire: a cross-national study. *Educ. Psychol. Meas.* 66 701–716. 10.1177/0013164405282471

[B65] Song Y. Gao J. (2020). Does telework stress employees out? A study on working at home and subjective well-being for wage/salary workers. *J. Happiness Stud.* 21 2649–2668. 10.1007/s10902-019-00196-6

[B66] Stempel C. R. Siestrup K. (2022). Suddenly telework: job crafting as a way to promote employee well-being? *Front. Psychol.* 12:790862. 10.3389/fpsyg.2021.790862 35095676 PMC8795870

[B67] Suh A. Lee J. (2017). Understanding teleworkers’ technostress and its influence on job satisfaction. *Internet Res.* 27 140–159. 10.1108/IntR-06-2015-0181

[B68] Tang M. Hofreiter S. Reiter-Palmon R. Bai X. Murugavel V. (2021). Creativity as a means to well-being in times of covid-19 pandemic: results of a cross-cultural study. *Front. Psychol.* 12:601389. 10.3389/fpsyg.2021.601389 33767644 PMC7985536

[B69] Taskin L. Devos V. (2005). Paradoxes from the individualization of human resource management: the case of telework. *J. Bus. Ethics* 62 13–24. 10.1007/s10551-005-8710-0

[B70] The jamovi project (2021). *jamovi (Version 2.2.2.0) [Computer Software]*. Available online at: https://www.jamovi.org

[B71] Tierney P. Farmer S. M. (2002). Creative self-efficacy: its potential antecedents and relationship to creative performance. *Acad. Manage. J.* 45 1137–1148. 10.2307/3069429

[B72] Udayar S. Urbanaviciute I. Massoudi K. Rossier J. (2020). The role of personality profiles in the longitudinal relationship between work–related well–being and life satisfaction among working adults in Switzerland. *Eur. J. Personal.* 34 77–92. 10.1002/per.2225

[B73] Vayre E. (2019). Les incidences du télétravail sur le travailleur dans les domaines professionnel, familial et social. *Trav. Hum.* 82:1. 10.3917/th.821.0001 18052372

[B74] Vayre E. Vonthron A.-M. (2019). Identifying work-related internet’s uses—at work and outside usual workplaces and hours—and their relationships with work–home interface, work engagement, and problematic internet behavior. *Front. Psychol.* 10:2118. 10.3389/fpsyg.2019.02118 31681056 PMC6797624

[B75] Vega R. P. Anderson A. J. Kaplan S. A. (2015). A within-person examination of the effects of telework. *J. Bus. Psychol.* 30 313–323. 10.1007/s10869-014-9359-4

[B76] Vittersø J. Akselsen S. Evjemo B. Julsrud T. E. Yttri B. Bergvik S. (2003). Impacts of home-based telework on quality of life for employees and their partners. Quantitative and qualitative results from a European survey. *J. Happiness Stud.* 4 201–233. 10.1023/A:1024490621548

[B77] Volk A. A. Brazil K. J. Franklin-Luther P. Dane A. V. Vaillancourt T. (2021). The influence of demographics and personality on COVID-19 coping in young adults. *Pers. Individ. Differ.* 168:110398. 10.1016/j.paid.2020.110398 32952250 PMC7492069

[B78] Waizenegger L. McKenna B. Cai W. Bendz T. (2020). An affordance perspective of team collaboration and enforced working from home during COVID-19. *Eur. J. Inf. Syst.* 29 429–442. 10.1080/0960085X.2020.1800417

[B79] Wang B. Liu Y. Qian J. Parker S. K. (2021). Achieving effective remote working during the covid-19 pandemic: a work design perspective. *Appl. Psychol.* 70 16–59. 10.1111/apps.12290 33230359 PMC7675760

[B80] Wang J. Cheng G. H. L. Chen T. Leung K. (2019). Team creativity/innovation in culturally diverse teams: a meta-analysis. *J. Organ. Behav.* 40 693–708. 10.1002/job.2362

[B81] Wang W. Albert L. Sun Q. (2020). Employee isolation and telecommuter organizational commitment. *Empl. Relat. Int. J.* 42 609–625. 10.1108/ER-06-2019-0246

[B82] Wilmot M. P. Wanberg C. R. Kammeyer-Mueller J. D. Ones D. S. (2019). Extraversion advantages at work: a quantitative review and synthesis of the meta-analytic evidence. *J. Appl. Psychol.* 104 1447–1470. 10.1037/apl0000415 31120263

[B83] Windeler J. B. Chudoba K. M. Sundrup R. Z. (2017). Getting away from them all: managing exhaustion from social interaction with telework. *J. Organ. Behav.* 38 977–995. 10.1002/job.2176

[B84] Wright S. L. Burt C. D. B. Strongman K. T. (2006). Loneliness in the workplace: construct definition and scale development. *N. Z. J. Psychol.* 35 59–68.

[B85] Zecca G. Györkös C. Becker J. Massoudi K. de Bruin G. P. Rossier J. (2015). Validation of the French Utrecht Work Engagement Scale and its relationship with personality traits and impulsivity. *Eur. Rev. Appl. Psychol.* 65 19–28. 10.1016/j.erap.2014.10.003

[B86] Zhou X. (2018). A review of researches workplace loneliness. *Psychology* 09 1005–1022. 10.4236/psych.2018.95064

