# Supplementary material for: Work-From-Home During COVID-19 Lockdown: When Employees’ Well-Being and Creativity Depend on Their Psychological Profiles
Source: Front Psychol. 2022 May 9;13:862987. doi: 10.3389/fpsyg.2022.862987 (PMC9126181; doi:10.3389/fpsyg.2022.862987)
Supplement: Supplementary file 1 [file Table_1.DOCX]

Supplementary Material

**Items of the scales used**

**Preference for Solitude Scale**

*For each of the following pairs of statements, select the one that best describes you. In some cases neither statement may describe you well or both may describe you somewhat. In those cases, please select the statement that best describes you or that describes you more often.*

1. a. I enjoy being around people.

b. I enjoy being by myself.

2. a. I try to structure my day so that I always have some time to myself.

b. I try to structure my day so that I always am doing something with someone.

3. a. One feature I look for in a job is the opportunity to interact with interesting people.

b. One feature I look for in a job is the opportunity to spend time by myself.

4. a. After spending a few hours surrounded by a lot of people, I usually find myself

stimulated and energetic.

b. After spending a few hours surrounded by a lot of people, I am usually eager to get

away by myself.

5. a. Time spent alone is often productive for me.

b. Time spent alone is often time wasted for me.

6. a. I often have a strong desire to get away by myself.

b. I rarely have a strong desire to get away by myself.

7. a. I like to vacation in places where there are a lot of people around and a lot of activities

going on.

b. I like to vacation in places where there are few people around and a lot of serenity and quiet

8. a. When I have to spend several hours alone, I find the time boring and unpleasant.

b. When I have to spend several hours alone, I find the time productive and pleasant.

9. a. If I were to take a several-hour plane trip, I would like to sit next to someone who was pleasant to talk with.

b. If I were to take a several-hour plane trip, I would like to spend the time quietly.

10. a. Time spent with other people is often boring and uninteresting.

b. Time spent alone is often boring and uninteresting.

11. a. I have a strong need to be around other people.

b. I do not have a strong need to be around other people.

12. a. There are many times when I just have to get away and be by myself.

b. There are rarely times when I just have to get away and be by myself.

*Note*. Items are reprinted from “Individual differences in preference for solitude” by Burger, 1995, *Journal of Research in Personality, 29*, 85-108. Copyright 1995 by Elsevier

**Personality variables**

*Here is a list of adjectives that may or may not fit you. For each proposal, indicate to what extent you agree or disagree.*

I see myself as someone who:

1. is reserved
2. tends to criticize others
3. works conscientiously
4. is 'relaxed', manages stress well
5. has a great imagination
6. is sociable, extrovert
7. usually trusts others
8. tends to be lazy
9. is easily upset
10. has little interest in anything artistic

*Note*. Items are reprinted from “Validation française du Big Five Inventory à 10 items (BFI-10)”, by Courtois, Petot, Plaisant, Allibe, Lignier, Réveillère, Lecocq, & John, 2020, *L'Encéphale*, 46(6), 455-462. Copyright 2020 by Elsevier

**Stress**

Stress means a situation in which a person feels tense, restless, nervous or anxious or is unable to sleep at night because his/her mind is troubled all the time. Do you feel this kind of stress these days?

**Loneliness at work**

| *Items relating to emotional deprivation* |
| --- |
| 1- I often feel abandoned by my colleagues when I am under pressure at work |
| 2- I often feel distant from my colleagues |
| 3- I feel removed from the people I work with |
| 4- I often feel emotionally distant from the people I work with |
| 5- I feel satisfied with the relationships I have at work* |
| 6- There is a sense of 'camaraderie' in my workplace* |
| 7- I often feel isolated when I am with my colleagues |
| 8- I often feel disconnected from others at work |
| 9- I experience a general sense of emptiness when I am at work |
| *Items related to social companionship* |
| 10- I have social companionship/fellowship at work* |
| 11- I participate in work-related social events* |
| 12- At work, there is someone I can talk to about my daily worries if I feel the need* |
| 13- There is no one at work with whom I can share my personal thoughts |
| 14- I have someone at work I can take my break with, if I want to* |
| 15- I feel part of a group of friends at work* |
| 16- There are people at work who take the trouble to listen to me* |

*Note*. Items are reprinted from “Loneliness in the workplace: Construct definition and scale development” by Wright, S. L., Burt, C. D. B., & Strongman, K. T., 2006, New Zealand Journal of Psychology, 35(2), 59–68. Copyright 2006 by American Psychological Association

**Job satisfaction**

Overall, I am satisfied with my work

**Work engagement**

1. At my work, I feel bursting with energy
2. At my job, I feel strong and vigorous
3. I am enthusiastic about my job
4. My job inspires me
5. When I get up in the morning, I feel like going to work
6. I feel happy when I am working intensely
7. I am proud of the work that I do
8. I am immersed in my work
9. I get carried away when I’m working

*Note*. Items are reprinted from “Validation of the French Utrecht Work Engagement Scale and its relationship with personality traits and impulsivity” by Zecca, Györkös, Becker, Massoudi, de Bruin, & Rossier, 2015, *European Review of Applied Psychology*, 65(1), 19-28, Copyright 2015 by Elsevier.

**Subjective creativity**

1. I have confidence in my ability to solve problems creatively
2. I have a certain talent for advancing the ideas of others
3. I think I am good at coming up with new ideas

*Note*. Items are reprinted from “Creative Self-Efficacy: Its potential antecedents and relationship to creative performance” by Tierney & Farmer, 2002, *The Academy of Management Journal,* *45*(6), 1137-1148. Copyright 2002 by Academy of Management.
